# Resilience, Life Events, and Well-Being During Midlife: Examining Resilience Subgroups

**DOI:** 10.1007/s10804-018-9288-y

**Published:** 2018-02-01

**Authors:** Debra McGinnis

**Affiliations:** Department of Psychology, Oakland University, Rochester, MI 48309 USA

**Keywords:** Midlife, Resilience, Life events

## Abstract

Developmental midlife processes involve resilience, changing challenges, and perceptions of getting older. In Study I and II, the Managing Life Survey resulted in growth, managing uncertainty, objectivity, adversity, and strategy use subscales. In Study II, resilience subgroups were identified. High and non-religious resilience groups had significantly higher averages for MLS subscales, time orientations, grit, life satisfaction; and significantly lower averages for adversity and negative event scores, compared to other groups. Noteworthy findings herein consist of (1) differences across resilience groups, with spiritual strategies emerging as an important discriminator; (2) the role of future perspectives on well-being characterizing early midlife; (3) the influence of growth and purpose on well-being characterizing late midlife; and (4) the cumulative effect of education on life satisfaction in late midlife. The results herein are consistent with the psychological benefits of moderate levels of challenge; with developmental differences across early and late midlife, and with Socioemotional Selectivity Theory.

## Introduction

Broadly speaking, psychological resilience is conceptualized as positive psychological adaptation after some degree of adversity (Bonano 2012; Masten 2001, 2014; Ong et al. 2009; Seery 2011). Lachman (2015) suggests that midlife may be characterized by peak levels of experiential learning and mature, but not-yet-declining cognitive abilities: characteristics that have the potential to lead to complex and variable, but not well-understood, developmental trajectories (also see Aldwin et al. 1996; and Blanchard Fields and Coats 2008). Most middle-aged adults cope with adversity competently, even though prior negative experiences and current stressors can present substantial challenges (American Association of Retired Persons, AARP 2001; Baltes 1987). Developmental processes during midlife not only pertain to the management of past and current circumstances, but also to some level of awareness of future age-related problems, possibly resulting in a unique transitional phase for many adults. Hence, midlife may be a *pivotal period* for many adults, characterized by heightened awareness of the meaning of past challenges, as well as preparing for future uncertainty (Aldwin and Levenson 2001; Heckhausen 2001; Lachman et al. 2015; Ryff 1991; Ryff and Singer 2003).

### Understanding Resilience in Adulthood

Among psychological scientists, conceptualizations of resilience vary. Some scientists studying resilience in adulthood have examined eventual reduction in psychopathology after adversity, empirically demonstrating reductions in post-event depression, anxiety, and post-traumatic stress symptomology (Fuller-Iglesias et al. 2008; Lucas 2007). Also focused on post-event outcomes is research that conceptualizes resilience as return to pre-event levels of well-being and life satisfaction (Lucas 2007; Seery et al. 2010). Hence, these resilience scientists have operationalized resilience as recovery to pre-event levels of well-being as well as reductions in psychopathology. Ryff and Singer (2003) propose that concepts of resilience are more thoroughly fleshed out when they include the idea of flourishing or thriving, suggesting that resilience and flourishing are related to “successful engagement with difficult events and experiences,” highlighting the role that managing challenges plays in this developmental process (Ryff and Singer 2003, p. 21).

Growth from particularly adverse experiences in adults has also been addressed in post-traumatic growth research (Tedeschi and Calhoun 2004). Post-traumatic growth refers to psychological growth that follows events that have disrupted self-perceptions and worldviews, with changes thought to influence personality change (Jayawickreme and Blackie 2014; Tedeschi and Calhoun 2004). Even though positive psychological change after trauma is theoretically and empirically significant, it may be more common to experience a variety of challenges in adulthood, rather than one or two major traumatic events (Damian and Roberts 2014; Seery et al. 2010; Seery and Kondrak 2014).

Whereas the child and adolescent resilience literature has evolved to include studies that address definitions, correlates, the nature of positive adaptation, interventions, and even neurobiological processes (Masten 2014; Wright et al. 2013), the adult development resilience literature is not as substantial. The study herein was designed to examine the nature, varieties, and covariates of resilience in midlife adults, potentially highlighting how midlife resilience compares to or differs from resilience at younger ages.

### Negative Life Experiences

Lucas et al. (2004) examined the psychological impact of particularly negative life experiences (e.g., disability, unemployment, spousal death) using large-scale panel studies (also see Lucas 2007). The patterns of recovery varied according to the type of experience. Individuals experiencing unemployment typically experience dramatic and immediate reductions in well-being, with notable recovery in the first year, but potentially incomplete recovery for many, even after several years. Individuals reporting disability (severe and moderate) also experience immediate and drastic reductions in well-being, with recovery being slow and potentially incomplete, particularly for those having experienced severe disability. A slightly different pattern characterizes recovery after death of a spouse which is also associated with declines in well-being, gradual recovery overall (about 7 years), and potentially incomplete recovery.

A second approach to the influence of negative life experiences involves the comparison of people with higher quantities of cumulative lifetime adversity to those with moderate and low levels, using a measure that sampled 37 adverse events (Seery et al. 2010). People reporting moderate levels of adversity (scores of 1–9) were more likely to score higher on well-being and lower on measures of negative psychological outcomes compared to people with scores of 10 or more. Participants with zero adversity were also lower on well-being and higher on measures of negative psychological outcomes, but these differences were smaller. Seery et al.’s (2010) results suggest that some adversity may be desirable, too much is undesirable, and that zero adversity may not be as psychologically negative as too much.

Seery et al.’s (2010) analysis did not address positive or negative perceptions associated with life events or event timing (e.g., young adulthood or middle adulthood). Even though it is safe to assume that events comprising their life event measure were considered negative, it is possible that some participants may not have viewed these experiences as particularly negative (e.g., a challenging divorce that ends a troubled marriage). Studies that enable the rating of life event negativity should decrease statistical noise when summing negative events, enabling increased sensitivity to patterns associated with adjustment processes and outcomes.

### Negative Life Events During Midlife

From a developmental perspective, psychological outcomes are influenced by the timing of events people experience as well as by the type of event. A taxonomy of life events, theoretically proposed by Baltes in 1987 and widely accepted by lifespan developmental scientists, includes age-graded, history-graded, and non-normative influences (Baltes 1987; Baltes and Smith 2004). Age-graded influences are those that occur around the same age for most individuals. History-graded influences are events that happen to everyone during a particular time frame. Non-normative influences are more idiosyncratic, with possibly no connection to age or historical timing (Baltes and Smith 2004). Adult development, according to Baltes and Smith (2004), is influenced by all of these, with non-normative influences contributing to variability across individuals.

Although most non-normative negative experiences can occur at any age, increases in particular ones characterize midlife. Middle-aged adults may experience physical problems for the first time (e.g., arthritis, hypertension) and age-related diseases may emerge. Problems related to work, relationships, and the loss of loved ones to death may increase. Variability across individuals in the types and quantities of negative non-normative experiences contribute to diverse trajectories, resulting in empirical and theoretical complexity (Baltes 1987; Lachman 2004). In addition, *early* midlife adults and *late* midlife adults may differ in the types of experiences they report (Stewart and Torges 2004; Stewart and Vandewater 1999). The transition to late life, with concerns about health and loss, against a backdrop of a lifetime of experiences, sets the stage for a unique and not well-understood phase of adulthood (Lachman 2015).

### Resilience and Well-Being in Midlife

As middle-aged or older adults experience or consider the negative effects of age-related challenges, it is natural to assume that well-being and life satisfaction would decline. However, developmental scientists report relative stability in well-being in adults under 70 years of age (Baird et al. 2010; Diener and Suh 1998; Kunzmann et al. 2000; Lachman et al. 2015; Mroczek and Spiro 2005). Alternatively, economic scientists report that well-being is at lowest point in midlife (Blanchflower and Oswald 2008; Frijters and Beatton 2012). These inconsistent results highlight the need for more research on well-being and its correlates across the life span.

Socioemotional Selectivity Theory (SST) and the empirical evidence supporting SST mechanisms provide justification for higher than expected levels of well-being in mid- and late life (Carstensen 1992; Carstensen et al. 1999). Reasonably successful management of adversity during midlife may be motivated by maximizing social, emotional, and psychological outcomes due to increasing awareness of finitude, as proposed by SST scientists (Carstensen 2009; Carstensen et al. 2003).

As middle-aged adults manage their sometimes unique and complex configurations of adversities, adversity management abilities may improve. It is also possible that the passage of time softens the impact that negative life events, increasing the tendency to reinterpret past events in a more positive manner (Infurna and Luthar 2016). In addition, psychological resilience could be severely impacted if problems are particularly severe, or if there are too many negative events in a compressed time frame.

Adults also differ in strategy preferences for managing adversity. For example, some adults may prefer religious- or spiritual-oriented strategies when facing challenges (AARP 2002; Manning 2013). Others may focus on humor as a strategy to deflect focusing on particularly negative aspects of these experiences and the consequences of these experiences (Marziali et al. 2008).

### Measuring Resilience

In the last couple of decades, several resilience scales have been developed, highlighting different conceptualizations of resilience. The Connor-Davidson Resilience Scale (CD-RISC, 25-items) was developed to study coping and self-efficacy in clinical populations (Connor and Davidson 2003). Additional studies substantiated the psychometric and conceptual utility of a shorter 10-item version (CD-RISC10) emphasizing coping and goal orientation (Campbell-Sills and Stein 2007).

Smith et al. (2008) pointed out that the CD-RISC subscales were related primarily to *resources* required for adaptation. Appropriately, their 6-item Brief Resilience Scale (BRS) addresses perceptions of recovery, yielding convergent validity with the CD-RISC, as well as unique variability (Smith et al. 2008).

Wagnild and Young (1993) developed and tested a 25-item scale for use in older adults, initially called *The Resilience Scale*. Factor analysis of this scale yielded two factors: self-efficacy and acceptance: abilities that may contribute to resilience, but are not associated with resilience as adaptation, recovery, or thriving.

Another approach to resilience examines how adults manage daily stress, with particular emphasis on how positive emotions buffer potentially negative outcomes after adversity with an emphasis on self-concept and control as buffers (Diehl and Hay 2010; Ong et al. 2009). Even though effective daily stress management reflects adaptation, it may be less related to overall recovery or thriving aspects of resilience. Taken together, these studies demonstrate that some empirical approaches address adaptation, whereas others address factors associated with adaptation. Continuing to clarify these differences conceptually and operationally would further the study of resilience in adults.

Among the goals of the present study was to develop a measure highlighting factors associated with positive adaptation after facing adversity, including growth and thriving. In addition, this measure included approaches or strategies often used when managing challenges; thereby focusing on processes as well as outcomes.

### Goals of Study I and Study II

The goals of the first study were to examine the utility of the Managing Life Survey (MLS) addressing perceptions of growth and purpose; managing uncertainty; strategy use; and adversity level. Because resilience has many connotations, the term resilience was not used in the survey name to avoid demand characteristics associated with conceptualizations of resilience.

The goals of second study included continued examination of the psychometric robustness and factor structure of the MLS. The Brief Resilience Scale (BRS) was included because the MLS did not include recovery items. A measure of persistence when encountering challenges (Grit) was also included (Duckworth and Quinn 2009). Together, the MLS with its growth and managing uncertainty scales, the BRS with its emphasis on recovery, and Grit measuring persistence in the face of challenges, comprise several important components of resilience. In addition, Carstensen and Lang’s (1996) future time perspective scale (FTP) was included to examine the relationships among measures of future orientation, resilience, and life events. The Holmes and Rahe (1967) live event survey (LES) was updated for use in this study by adding items related to unemployment, abuse, and bullying. Participants also were asked if selected life events were among their five worst experiences.

In short, the goals of Study II were (1) to examine resilience broadly and identify correlates of measures of resilience; (2) to obtain negative life event counts in a middle-aged sample to identify which events were considered most negative, how often these occurred to participants, and in which stage of life these events occurred (e.g., childhood, young adulthood); (3) to identify resilience subgroups using the MLS; (4) to examine negative life event counts across resilience subgroups; (5) to examine additional characteristics of these subgroups (e.g., age, education, recovery, perceptions of future opportunities, and persistence); and (6) to examine the influence of negative life events and measures of resilience on overall well-being.

## Study I: Validation of the Managing Life Survey

### Method

#### Participants

Participants were recruited from Amazon’s Mechanical Turk (AMT) and provided a link to the survey in Survey Monkey. Participants were informed that minimum requirements consisted of U.S. residence, English as a first language, and a minimum age of 30. The quantity of participants was monitored with the goal of obtaining a sample balanced across four age groups (30-year olds, 40, 50, and 60 +). To evaluate attention to content, participant responses on three pairs of matched survey questions (questions that asked for nearly the same information) were evaluated for consistency. Participants whose responses were inconsistent were eliminated. The final sample consisted of 358 participants (190 males, 165 females, 3 missing gender). The age range was 30–80 years of age (*M* = 47.8, SD = 12.57). The average education in years was 15.17 (SD = 2.33), and degree attainment frequencies were 169 for HS; 128, BA; and 59, MA/PhD; with 2 not completing high school.

### Materials and Procedures

#### Background

Participants were asked to indicate age, gender, and years of education.

#### Managing Life Survey (MLS)

Survey questions addressing various aspects of resilience were developed. MLS statements (77) addressed growth and purpose; managing uncertainty and life problems; emotional objectivity; humor; spirituality; changing philosophy with age; persistence; and level of adversity during different age ranges (e.g., 20–39; 40–59). Participants rated each statement using a 6-point scale (*1* = *disagree absolutely to 6* = *agree absolutely*). Reverse-scored items were randomly dispersed.

#### Procedure

Participants answered the background questions and responded to the MLS after responding to the informed consent. After completion, participants returned to AMT and entered a code to enable payment. Participation took about 10 min and participants were paid $1.00 each (reported as reasonable on the AMT participant forum).

#### MLS Missing Data

Out of a possible 27,566 responses, 138 responses were missing, yielding a 0.5% missing data rate. Because this is a small percentage, mean replacement was used for missing data.

## Results and Discussion

### Factor and Reliability Analyses of MLS

Factor analysis of the 77 item MLS using principal component analysis and varimax rotation was performed. Because some participants were under 40, two questions addressing hardship in late life were not included in the analysis. Data reduction proceeded by eliminating from the rotated component matrix all of the items loading alone, followed by conceptually inconsistent items loading on the final two factors, followed by removal of factors loading across two or more items with the secondary (second highest) above .35. This process resulted in a seven-factor solution accounting for 70.6% of the variance. Table 1 includes the factor and reliability statistics.

**Table 1:** Study 1—MLS factors and factor loadings

	Factor						
	1	2	3	4	5	6	7
1. Growth/purpose							
MLS-39	.811						
MLS-48	.777						
MLS-77	.765						
MLS-13	.735						
MLS-25	.730						
MLS-56	.729						
MLS-04	.712						
MLS-68	.683						
MLS-59	.636						
2. Managing uncertainty							
MLS-23		.752					
MLS-30		.728					
MLS-44		.726					
MLS-53		.697					
MLS-74		.675					
MLS-5		.655					
MLS-17		.642					
3. Spirituality							
MLS-37R			.945				
MLS-63			.942				
MLS-60			.934				
MLS-19			.926				
4. Adversity level							
MLS-35				.853			
MLS-7				.813			
MLS-18				.804			
5. Emotional objectivity							
MLS-01		− .321			.807		
MLS-27		− .323			.792		
MLS-72R		− .424			.650		
6. Humor							
MLS-66						.855	
MLS-51R						.853	
MLS-02R	.302					.549	
7. Changing philosophy							
MLS-15R							.908
MLS-22R							.905
Variance	17.77%	14.03%	12.11%	7.78%	6.61%	6.60%	5.70%
Cronbach’s *α*	.910	.859	.972	.837	.836	.817	.814

*N* = 358. Adversity items (two) related to adversity in adults over 40 were not included in this analysis due to missing data for participants over 40

Three factors accounted for over 10% of the variance each: growth, managing uncertainty, and spirituality. The remaining components (adversity, emotional objectivity, humor, and changing philosophy) accounted for between 5.7 and 7.7% of the variance each. Reliability analyses were conducted for these seven factors, with outcomes ranging from .81 to .97.

Factor and reliability analyses supported the use of a 31-item MLS instrument, with seven factors: growth and purpose; managing uncertainty; spirituality; emotional objectivity; adversity level; humor and changing philosophy. Two additional items addressing mid and late life adversity should be included when individuals over 60 are recruited, yielding a 33-item instrument.

### MLS Subscale Correlations

Table 2 includes the correlations among the seven resilience subscales. Growth was correlated with most subscales except Adversity and Change in Philosophy. Managing Uncertainty was also correlated with most of the subscales, except Spirituality and Change in Philosophy.

**Table 2:** Study 1—MLS subscale correlations

	2	3	4	5	6	7
1. Growth	− .347***	.357***	.078	.304***	.497***	.036
2. Man Uncer	–	− .099	.316***	− .585***	− .443***	.072
3. Spirit		–	.005	− .061	.146**	− .067
4. Adv			–	− .188***	− .171***	.115*
5. Emo				–	.385***	− .115*
6. Humor					–	.006
7. Change						–

*N* = 358 for all correlations. *Man Uncer* managing uncertainty, *Spirit* spirituality, *Adv* adversity under 40, *Emo* emotional objectivity, *Change* change philosophy

*p < .05, **p < .01, ***p < .001

Even though growth, the management of uncertainty, and the role of spiritual strategies are important aspects of resilience, the measurement of resilience should also include measures that address recovery from hardship and persistence in the face of challenges. In addition, the possible relationships among life event quantities, life event types, and resilience variables should be explored. These issues are addressed in Study 2.

## Study II: Resilience and Life Events in Midlife

### Method

#### Participants

Participants were recruited from Amazon’s Mechanical Turk (AMT) and provided a link to the survey in Survey Monkey (*N* = 276). Before agreeing to participate, participants were informed that minimum requirements consisted of U.S. residence, English as a first language, and a minimum age of 45. Participant ages were monitored to enable a sample balanced across the age range.

Because it is less common for adults in their early 40s to think of themselves as middle-aged, a minimum age of 45 was set. In addition, it is not uncommon for people in their late 60s or early 70s to think of themselves as middle-aged due to feeling reasonably healthy overall (Lachman 2004). People over the age of 45 and under 76 were recruited. The 30-year time frame corresponds to early midlife, approximately 45–60; and late midlife, 60–75. The late midlife group overlaps the age range of “young old” in other studies, but given the lengthening of midlife in developed countries it can be said that this period can also be referred to as late midlife. A median split will be used for midlife groups.

After screening was complete, the sample consisted of 267 participants (130 males, 137 females). The age range was 45–75 years of age (*M* = 57.6, SD = 8.16). A median split resulted in an *early midlife* group under 58 years of age (*N* = 134) and a *late midlife* group 58 and older (*N* = 133). The average education in years was 15.06 (SD = 2.05). Overall, 114 had a HS education; 77 had attended but had not graduated from a four year program; 112 had earned a B.A.; 36, a M.A. or Ph.D.; and 5 participants reported a 10th grade education. Age group and education level (10th grade, HS, BA, MA/Ph.D.) were not associated, *χ*^*2*^(3, *N* = 267) = 5.94, *p* = .114.

### Materials and Procedure

1. Background Information: Age, gender, and years of education. To compute education variables, participants were asked to report highest level of high school or college completed or degree attained.
2. Modified Life Event Survey: Holmes and Rahe (1967) Life Event Survey (Social Readjustment Scale) was updated to include questions about unemployment, experiencing bullying or personal violence, and car accidents using. Following each selected event, participants were presented with these two statements: “This event had an extremely significant impact on my life”; and “When this event occurred, it was one of the five most negative experiences in my entire life”; and a 6-point scale (*1* = *disagree absolutely to 6* = *agree absolutely*); and by “Please indicate your approximate age when this event occurred,” (response options: 0–19; 20–39; 40–59; 60–79; and 80 +)
3. Managing Life Survey (MLS): The MLS consists of 33 items addressing several aspects of resilience (growth, managing uncertainty, spirituality, humor, emotional objectivity, adversity, and change with age), using a 6-point scale (*1* = *disagree absolutely to 6* = *agree absolutely)*.
4. Brief Resilience Scale (BRS): The BRS consists of 5-items addressing recovery aspects of resilience, using a 7-point scale, (*1* = *strongly disagree to 7* = *strongly agree*) (Smith et al. 2008).
5. Short Grit Scale (Grit): Grit is an 8-item instrument addressing diligence and focus on goals, with a 5-point scale, (*1* = *not like me at all to 5* = *very much like me*) (Duckworth and Quinn 2009)
6. Future Time Perspective (FTP): The FTP is a 10-item item scale addressing perceived opportunities in the future, with a 6-point scale (*1* = *very untrue to 6* = *very true*) (Carstensen and Lang 1996).
7. Subjective Well-being (SWL): Five item scale measuring overall satisfaction with life so far using a 7-point scale (*1* = *strongly disagree to 7* = *strongly agree*) (Diener et al. 1985).

#### Procedure

Participants answered the background questions and responded to the surveys. To minimize participant fatigue and inattention, format presentation varied across surveys (one item per page to a list of items on one page). To evaluate attention to content, participant responses on five screening questions were evaluated. Sample questions included “The answer for this item is slightly agree” and “I lost both of my arms in a sawmill accident.” Participant data for nine participants answering inaccurately were eliminated. After completion, participants returned to AMT and entered a code to enable payment of $1.50 (reported as sufficient on AMT worker forum). Participation took approximately 15 min with a range of 10–20 min.

### Data Analysis

Data analysis proceeded as follows: (1) factor and reliability analysis of all of the scales; (2) quantification of life events (positive, negative, age range); (3) effects of midlife group on negative life event counts; (4) identification and confirmation of resilience subgroups using cluster and discriminant analysis; (5) five (resilience group) by two (midlife group) MANOVAs for the variables FTP and SWL, and Grit; (6) five (resilience group) by two (midlife group) MANOVAs for life event timing categories (childhood, young adult, middle-age); (7) five (resilience group) by two (midlife group) MANOVAs for life event category variables (e.g., death totals, finances, etc.); (8) stepwise regression to examine the influence of resilience, life event variables, age, and education on life satisfaction with a final analysis including just the most robust predictors; and (9) ANOVA analyses of the effect of educational attainment on measures of adversity.

## Results

### Factor and Reliability Analyses of Scales

Factor analysis of the 33-item MLS using principal component analysis and varimax rotation was performed. Data reduction proceeded by eliminating items loading alone, followed by conceptually inconsistent items loading on the last factor, followed by removal of items loading across two or more factors with the secondary loading above .40. This process resulted in a seven-factor solution for 25 items resembling the factor solution obtained in Study 1. These seven factors accounted for 73.2% of the variance. Table 3 includes the factor and reliability statistics.

**Table 3:** Study 2—MLS factors and factor loadings

	Factor						
	1	2	3	4	5	6	7
1. Growth/purpose (7 items)							
MLS-7	.770						
MLS-14	.757						
MLS-5	.732						
MLS-36	.731						
MLS-1R	.713			.329			
MLS-21	.695						
MLS29	.665						
2. Spirituality (4 items)							
MLS-24		.944					
MLS-16R		.942					
MLS-31		.932					
MLS-6R		.917					
3. Adversity level (4 items)							
MLS-35			.875				
MLS-4			.788				
MLS-32			.779				
MLS-23			.742				
4. Managing uncertainty (4 items)							
MLS-9R				.793			
MLS-28R				.762			
MLS-22R				.737			
MLS-11R				.662			
5. Emotional objectivity (2 items)							
MLS-2					.846		
MLS-30					.823		
6. Change in philosophy (2 items)							
MLS-10R						.821	
MLS-26R						.814	
7. Humor (2 items)							
MLS-18R							.861
MLS-25	.335						.845
% Variance	16.87%	14.65%	11.09%	10.77%	6.85%	6.79%	6.18%
Cronbach’s *α*	.878	.966	.824	.815	.812	.656	.836

*N* = 267

Four factors accounted for over 10% of the variance each: growth, spirituality, adversity, and managing uncertainty. The remaining components (emotional objectivity, change in philosophy, and humor) accounted for between 5.7 and 7.8% of the variance each. Reliability analyses were conducted for these seven subscales, with all but one outcome over .80.

Factor and reliability analyses supported the use of a 25-item MLS instrument, with seven factors: growth and purpose (7 items); spirituality (4 items); managing uncertainty (4 items); adversity level (4 items); emotional objectivity (2 items); change in philosophy (2 items); and humor (2 items), replicating the outcome obtained in Study I. The 25-item MLS is presented in its entirety in Appendix, with reverse scored items noted and the item numbers from the survey as administered. Subscale averages were computed for each participant.

Reliability analyses for the remaining scales (BRS, Grit, FTP, and SWL) were robust, yielding Cronbach’s alphas similar to previous studies (BRS, .940; Grit, .867; FTP, .941; SWL, .916). Subscale averages were computed for each participant.

### Frequencies of Life Events

Table 4 provides the life event frequencies across the entire sample. The most frequent life event experienced was close family death (*N* = 234). Several life events occurred to over 100 participants. For example, 108 people experienced divorce; 124, personal injury; 111, getting fired; 170, family member health change; 152, major financial difficulties; 115, close friend death; 138, job change; 147, children leaving home; and 151, moving to another town or state. In the negative column, 200 out of 234 rated close family death as one of the most negative experiences; 50 out of 124, personal injury; 40 out of 111, getting fired; 105 out of 170, family member health change; 103 out of 152, major financial difficulties; 71 out of 115, close friend death; 29 out of 138, major job change; only 20 out of 147 for children leaving home; and only 16 out of 151 for moving to another town or state. In this sample, the experience of children leaving home or major moves out of town or state were not considered negative.

**Table 4:** Study 2—life event frequencies

Life events		Negative	Positive	Total
1.	Spouse death	32	1	35
2.	Divorce	72	13	108
3.	Marital separation	13	2	19
4.	Close family death	200	7	234
5.	Personal injury	50	36	124
6.	Serious illness	44	7	72
7.	Getting fired	40	43	111
8.	Family member health change	105	9	170
9.	Major financial difficulties	103	9	152
10.	Close friend death	71	7	115
11.	Major job change	29	83	138
12.	Worrisome marital discord	19	6	48
13.	Mortgage or loan foreclosure	15	2	30
14.	Work responsibility changes	12	56	86
15.	Children leaving home	20	80	147
16.	Trouble with relatives	22	27	83
17.	Moving to another town/state	16	107	151
18.	Negative changes in living conditions	34	12	71
19.	Trouble with boss or manager	17	48	95
20.	Neg changes in work hours/conditions	10	25	50
21.	Problems continuing recr activities	17	17	55
22.	Problems with rel/spiritual activities	3	2	11
23.	Problems with social activities	6	9	26
24.	Long-term unemployment	49	7	74
25.	Changes in sleeping habits	8	44	92
26.	Minor law violations	10	35	56
27.	Serious vehicular accident	21	14	56
28.	Vehicle repossession	4	10	23
29.	Parental divorce	24	18	53
30.	Victim of bullying at school or work	25	11	49
31.	Physical abuse during childhood	24	1	30
32.	Sexual abuse during childhood	24	1	30
33.	Victim of abusive rom relationship	29	3	47
34.	Victim of rape	10	0	14
35.	War trauma	12	1	15
36.	Major flood or fire disaster	16	6	31
37.	Serious life event—I	19	6	28
38.	Serious life event—II	3	1	4

Negative life events correspond to any event rated 5 or 6 for the statement “This event was one of the five most negative events in my life.” Positive life events correspond to those rated 1 or 2 on the same scale. The total includes positive, neutral (3, 4 on the scale), and negative life events

### Life Event Counts: Valence, Categories, Timing

Life event totals were computed for each participant. The average quantity of life events overall was 10.24 (SD = 4.89; min = 1; max = 25). In addition, each life event was coded as primarily negative (score of 5 or 6 on the most negative experience question) or primarily positive (score of 1 or 2). All other events were coded as neutral. Overall, participants reported an average of 4.60 negative life events (SD = 3.18; min = 0; max = 16), suggesting that less than half of the life events reported were perceived as negative.

Participants indicated whether each event occurred in childhood, young adulthood, middle adulthood, or after 60, yielding negative childhood, young adulthood, and middle adulthood counts. These are also included in Table 5. Participants reported more challenges in midlife compared to younger periods (*M* = 2.4 for midlife; *M* = 1.49 for young adulthood), and many did not even report one childhood event (M = .63). This outcome is not surprising, given that is relevant to challenges in adulthood.

**Table 5:** Study 2—negative life event frequencies: timing, categories

	0	1	2	3	4	5	6	*f* > 6	*M*	*SD*
Timing										
Childhood	164	63	24	11	3	1	1	0	0.63	0.99
Young adulthood	77	85	55	21	19	6	1	3	1.49	1.58
Middle adulthood	51	58	44	45	25	17	12	15	2.48	2.22
Total adulthood	9	39	49	46	26	29	20	49	3.97	2.77
Total NLE	6	32	39	45	30	29	21	65	4.60	3.18
Category										
Deaths	68	132	63	4	0	0	–	–	1.01	0.74
Relationship	159	89	16	3	0	0	–	–	0.49	0.66
Employment	178	46	25	14	3	1	–	–	0.58	0.98
Finances	162	90	15	0	0	0	–	–	0.45	0.60
Health issues	121	101	30	14	1	0	–	–	0.78	0.87
Abuse	232	33	2	0	0	0	–	–	0.14	0.37
Trauma	230	32	5	0	0	0	–	–	0.16	0.41

*N* = 267. Cell counts reflect the number of individuals with each frequency. For example, 63 participants reported one negative childhood life event, and 24 reported two negative childhood life events. Means below 0 arise when a substantial proportion report no life events in that category. For example, most participants did not report any childhood negative events

Using adulthood events rated negative, life event categories were computed: death, relationship, employment, finances, health, abuse, and trauma. Except for death totals, most participants had life event category scores of 0. Table 5 includes the frequencies of these categories across the entire sample.

### Midlife Group and Life Event Counts

Table 6 includes the significant differences across the frequencies of negative life events. Late midlife participants were more likely to experience spousal death than early midlife participants, but this difference was not quite statistical significant. Late midlife adults were also more likely to report more negatively rated close family deaths and war traumas: differences that were significant. None of the early midlife group reported negative war trauma. Early midlife adults had higher rates of negative divorces, losing their jobs, financial difficulties, long-term unemployment, and parental divorce: differences that were significant. Taken together, these data suggest that death events may be more common in late midlife, but that problems with relationships, employment, and finances are more common in early midlife. It is noteworthy that early and late midlife adults reported similar quantities of health problems.

**Table 6:** Study 2—negative life event counts by midlife group

	Midlife group		Statistic	Effect size
	Early	Late	*χ* ^*2*^	*ϕ*
Life event raw counts				
Spousal death	11	21	3.64^+^	.117
Divorce	46	26	7.40**	.167
Close family death	93	107	4.34*	.127
Getting fired	26	14	4.13*	.124
Major financial difficulties	66	37	12.94***	.220
Long-term unemployment	33	16	7.07**	.163
Parental divorce	17	7	4.50*	.130
War trauma	0	12	12.66***	.218
Life event categories			*t*(265)	*d*
Deaths	0.86	1.17	− 3.45***	.426
Relationship	0.57	0.41	1.99*	.244
Employment	0.75	0.41	2.94**	.368
Finances	0.57	0.32	3.49***	.429
Health issues	0.77	0.78	− 0.12	.015
Abuse	0.18	0.10	1.81	.226
Trauma	0.12	0.20	− 1.51	.185

*N* = 267 for all analyses. Life event raw data were dichotomized to examine differences across early and late midlife. For example, 11 of the early midlife group reported one spousal death. Life Event category data were not dichotomized, so *t* tests were used to examine the differences

*p < .05, **p < .01, ***p < .00, +*p* = .057

### Resilience Cluster Subgroups

Resilience variables (growth, spirituality, managing uncertainty, and humor) were used in a Quick Cluster analysis to examine cluster subgroups. Meaningful profiles were obtained for 4–6 cluster solutions. Table 7 includes the cluster center profiles for the 4-, 5-, and 6-cluster outcomes after deleting participants with outlying cluster center distance scores.

**Table 7:** Study 2—final cluster centers for four-, five-, and six-cluster outcomes

	Six-cluster outcome					
	Low-resilient 1	Low-resilient 2	Religious low humor	Medium	Non-religious	Resilient
Growth	2.57	3.95	4.10	4.03	4.51	4.95
Spirituality	1.51	1.62	5.38	3.71	1.56	5.34
Mang unc	2.47	2.98	3.21	3.51	4.46	4.84
Humor	1.58	3.77	2.54	3.92	4.89	4.83
*N* = 258	18	26	49	53	40	72

	Five-cluster outcome				
	Low-resilient	Religious	Medium	Non-religious	Resilient
Growth	2.76	4.09	3.89	4.35	4.87
Spirituality	1.46	5.38	3.17	1.52	5.20
Mang unc	2.53	3.20	3.34	4.17	4.68
Humor	1.65	2.58	3.71	4.74	4.77
*N* = 253	19	49	51	50	84

	Four-cluster outcome			
	Low-resilient	Religious	Non-religious	Resilient
Growth	2.98	4.02	4.21	4.92
Spirituality	1.63	5.03	2.07	5.23
Mang unc	2.61	3.16	3.89	4.78
Humor	1.89	3.12	4.36	4.82
*N* = 249	23	68	81	77

All of these cluster solutions included a profile with very high scores overall (resilients); a profile with very low scores overall (low resilients); and a profile with medium scores (medium resilients). The 5- and 6-cluster outcomes also included groups reflecting high and low spirituality, but differing on other variables, suggesting that these two profiles are conceptually meaningful (religious and non-religious). The 4-cluster solution began to diverge from the consistency observed for the 5- and 6-cluster solutions. It appears that the group in the middle was dispersed across other groups in the 4-cluster solution, possibly decreasing discriminatory precision. The 5-cluster solution provided an outcome that encompassed conceptually meaningful profiles observed in both the 5- and 6-cluster outcomes, preserving specificity.

Discriminant analysis was used to validate the five-cluster group membership classifications, beginning with the 253 participants remaining after eliminating cluster distance outliers. Discriminant analysis produced a consistent outcome, with the exception of 4 mis-predictions. After eliminating the mis-predicted cases, the sample sizes for the subgroups were as follows: Low Resilients: 19; Religious: 49; Medium Resilients: 51: Non-religious: 50, and High Resilients: 80, for a total of 249 participants.

**Fig. 1:**
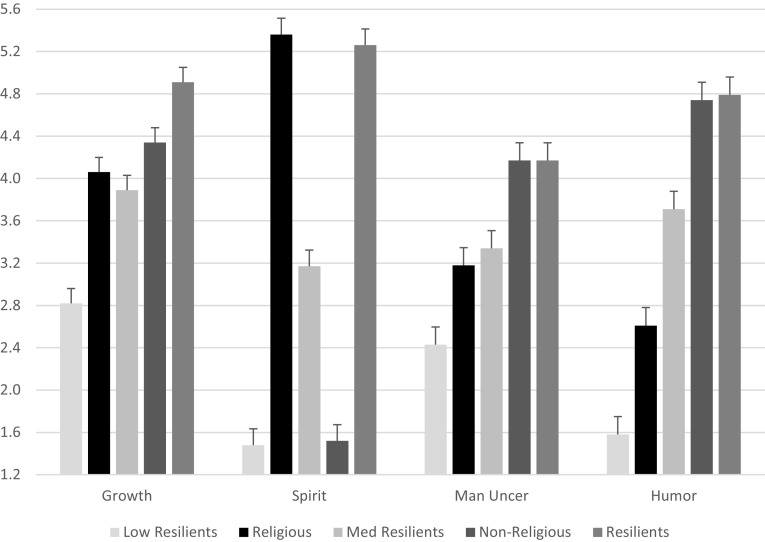
MLS subscale means across five subgroups. Note: *N* = 249 overall. Main effects of resilience subtype on all MLS subscale means. Error bar = + 1 SEM

As expected, omnibus ANOVA tests for the effects of resilience subgroups on growth, spirituality, managing uncertainty, and humor were significant: Growth, *F* (4, 248) = 45.59, *p* ≤ .001; Spirituality, *F* (4, 244) = 344.58, *p* ≤ .001; Managing Uncertainty, *F* (4, 244) = 54.27, *p* ≤ .001; and Humor, *F* (4, 244) = 70.83, *p* ≤ .001. Table 8 provides the means and ANOVA statistics, including post hocs. Figure 1 graphs the means for these variables across groups, depicting quantitative and qualitative differences.

**Table 8:** Study 2: descriptives for five resilience subgroups

	Low resilients	Religious	Medium resilients	Non-religious	Resilient	Total	*F*	*η* ^*2*^
	*N* = 19	*N* = 49	*N* = 51	*N* = 50	*N* = 80	*N* = 249	(4, 244)	
MLS								
Growth	2,82_ab_ (0.86)	4.06_a_ (0.69)	3.89_b_ (0.60)	4.34_ab_ (0.75)	4.90_ab_ (0.58)	4.26 (0.87)	45.59***	.428
Spirituality	1.48_ab_ (0.74)	5.36_ac_ (0.68)	3.17_abc_ (0.75)	1.52_c_ (0.60)	5.26_b_ (0.72)	3.81 (1.79)	344.58***	.850
Man uncer	2.43_ab_ (0.97)	3.18_a_ (0.74)	3.34_b_ (0.64)	4.17_ab_ (0.85)	4.17_ab_ (0.56)	3.85 (1.09)	54.27***	.471
Humor	1.58_ab_ (0.58)	2.61_ab_ (0.99)	3.71_ab_ (0.62)	4.74_a_ (0.74)	4.79_b_ (0.76)	3.88 (1.32)	118.45***	.660
Demographics								
Age	57.42 (7.06)	58.51 (7.89)	59.71_a_ (8.62)	55.90_a_ (6.99)	57.04 (8.65)	57.67 (8.12)	1.67	.027
Educ	14,37_a_ (2.22)	14.57_b_ (1.83)	15.18 (2.27)	15.10 (1.74)	15,41_ab_ (2.05)	15.06 (2.03)	1.95	.031
Adversity, resilience, and well-being								
Adversity	4.53_abc_ (0.99)	4.30_def_ (1.03)	3.82_ad_ (1.03)	3.54_be_ (1.21)	3.86_cf_ (1.12)	3.93 (1.13)	4.58***	.070
Objectivity	3.11_abc_ (1.08)	3.79_ad_ (1.10)	3.82_bc_ (1.03)	4.45_ab_ (0.82)	4.63_cd_ (0.85)	4.15 (1.04)	16.03***	.208
Chg in phil	4.21_abc_ (1.34)	4.01_c_ (1.30)	3.47_bc_ (1.01)	3.92_b_ (1.30)	4.01_c_ (1.36)	3.90 (1.28)	1.99	.032
BRS	3.33_ab_ (1.04)	4.00_a_ (0.93)	4.16_b_ (0.75)	4.80_ab_ (0.70)	5.03_ab_ (0.62)	4.48 (0.93)	29.95***	.329
Grit	4.26_ab_ (0.81)	4.54_c_ (0.72)	4.53_d_ (0.56)	4.78_b_ (0.64)	4.93_acd_ (0.68)	4.69 (0.69)	6.03***	.090
FTP	1.97_ab_ (0.67)	3.01_a_ (0.98)	3.37_b_ (0.86)	3.48_a_ (1.00)	4.10_ab_ (1.14)	3.45 (1.15)	21.44***	.260
SWL	2.58_abc_ (1.29)	4.08_ab_ (1.55)	4.36_c_ (1.26)	4.86_a_ (1.30)	5.21_bc_ (1.11)	4.54 (1.47)	18.89***	.236
Life events (negative, positive)								
Neg LE	5.84_ab_ (4.75)	5.37_cd_ (3.72)	3.71_ac_ (2.44)	3.96_bd_ (2.32)	4.53 (3.02)	4.51 (3.16)	3.05*	.048
Pos LE	2.63 (2.69)	2.51 (2.17)	2.43 (1.93)	3.28 (2.73)	3.14 (2.67)	2.86 (2.46)	1.30	.021

SDs in parentheses. Matching subscripts indicate significant post hoc results. For education, raw education levels were used: 11 = did not finish HS; 12 = HS; 13 = HS + 1 year college; 14 = HS + 2; 15 = HS + 3; 16 = BA; 17 = MA; 18 = Ph.D.

*p < .05, **p < .01, ***p < .001

It is possible that these resilience groups characterize people in the following ways. Participants classified as high resilients (*N* = 80) have the highest scores overall. This is also the largest cluster suggesting that middle-aged people may manage adversity reasonably well, may experience a sense of growth and purpose from these accomplishments, and are engaging spiritually in a generally effective manner. The MLS means for the non-religious group (*N* = 50) are almost as high as the high resilients, suggesting that these participants perceive that they are able to face life’s challenges without the use of spiritual or religious strategies. The medium resilient group falls around subscale averages. The religious subgroup participants (*N* = 49) have higher MLS subscale scores endorsed the use of religious or spiritual strategies with lower scores on other MLS subscales. The scores for the participants in the low resilient group (*N* = 19) reflect remarkably low levels across all of these resilience variables, possibly due to extreme hardship or diminished abilities to manage life circumstances.

### Resilience Group Analyses: Demographics, Adversity, BRS, Grit, FTP, and SWL

Table 8 also includes the means for age, education, Adversity, BRS, Grit, FTP, and SWP, negative life events, and positive life events. Resilience groups did not differ statistically for the variables age or education. However, about two-thirds of the non-religious group (66%) had earned at least a 4-year degree compared to just under one-third of the low resilient group (31%). MLS-adversity means were lowest in the non-religious group and highest in the low resilient and the religious groups.

**Fig. 2:**
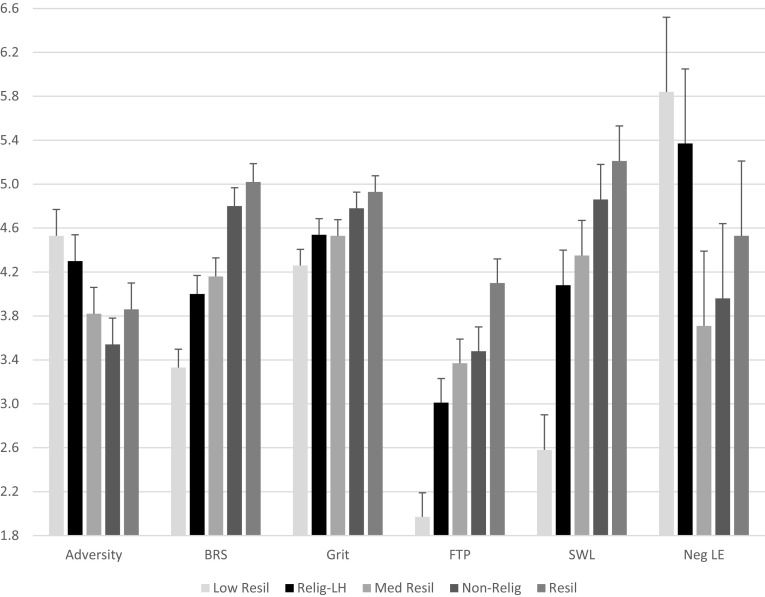
Resilience variable means across resilience subgroups. Note: *N* = 249. Error bars = standard errors for comparisons across the five subgroups. Main effects for resilience group on adversity, BRS, Grit, FTP, SWL, and Neg LE were all significant. Error bar = + 1 SEM

Five (resilience groups) by two (midlife groups) repeated measures MANOVA analyses were conducted for the following dependent variables: FTP, BRS, SWL, and Grit. Mauchly’s test of sphericity indicated that the covariance matrices were not homogenous, but the sphericity-assumed within-subjects statistics were similar to the adjusted statistics (Greenhouse-Geisser correction factor was .924). Greenhouse-Geisser adjusted within-subjects statistics are provided. The multivariate within-subjects outcome across the standardized resilience measures was not significant. The resilience group by resilience measures multivariate interaction was significant, *F* (12, 717) = 2.34, *p* = .008,* η*^*2*^ = .038: an outcome that reflects the preponderance of higher scores for the non-religious and the high resilient groups. Table 8 and Fig. 2 provide the means for these groups.

The within-subjects midlife group by resilience measure type interaction was significant, *F* (3, 717) = 3.66, *p* = .015, * η*^2^ = .015, due to higher FTP for early midlife adults compared to late midlife adults (*M* = 3.61, 3.29; *SD* = 1.22, 1.06, respectively); and much lower SWL (*M* = 4.36, 4.73; *SD* = 1.54, 1.37, respectively). The within-subjects three-way interaction (resilience measures by resilient group by midlife group) was not significant. The between subjects effect of resilience group was also significant, *F* (4, 239) = 36.40, *p* ≤ .001 *η*^2^ = .379, but the between subjects effect of midlife group was not significant, nor was the between subjects midlife group by resilient group interaction. Significant between-subjects effects were obtained as follows: FTP: *F* (4, 239) = 20.65, *p* ≤ .001, *η*^2^ = .257; BRS: *F* (4, 239) = 29.45, *p* ≤ .001, *η*^2^ = .330; SWL: *F* (4, 239) = 19.77, *p* ≤ .001, *η*^2^ = .249, and Grit: *F* (4, 239) = 6.37, *p* ≤ .001, *η*^2^ = .096.

### Resilience Group Analyses: Life Event Variables

#### Life Event Timing

Five (resilient groups) by two (midlife groups) repeated measures MANOVA analyses were also conducted for negative life event timing variables: childhood, young adulthood, and middle adulthood. Because the levels of negative childhood experiences were very low (most participants reported none), this analysis was repeated without that level, resulting in two levels of life event timing (young and middle adulthood). Participants reported more negative life events from the ages of 40–60 compared to earlier time frames, (*M* = 1.45, 2.44; *SD* = 1.56, 2.18); *F* (1, 239) = 31.81, *p* ≤ .001 *η*^2^ = .117. Neither the interaction with resilient group or midlife group was statistically significant, but the three-way interaction (LE timing by resilient group by midlife group) was significant, *F* (4, 239) = 2.95, *p* = .021, *η*^2^ = .047. Contributing to this significant difference was the much larger quantity of negative middle-age experiences for the low resilient early midlife participants compared to the low resilient late midlife participants (*M* = 4.50, 1.67; *SD* = 2.17, 3.15, respectively). The between subjects effect of midlife group was significant, *F* (1, 239) = 4.67, *p* = .032, *η*^2^ = .019, with more negative life experiences reported by the early midlife group (*M* = 4.90. 4.12; *SD* = 3.57, 2.65, respectively). The between subjects effect of resilient group was also significant, *F* (4, 239) = 2.92, *p* = .022, *η*^2^ = .047 due to the greater quantity of negative life events reported by the low resilient and religious participants. Life event timing means are provided in Table 9.

**Table 9:** Negative life event counts by resilient and midlife groups

	Low resilients	Religious	Medium resilients	Non-religious	Resilient	Total
	*N* = 19	*N* = 49	*N* = 51	*N* = 50	*N* = 80	*N* = 249
Neg LE timing						
Young adulthood events						
Early midlife	1.80 (1.99)	2.56 (2.44)	1.50 (1.50)	1.41 (1.31)	1.75 (1.38)	1.79 (1.70)
Late midlife	1.33 (1.11)	1.27 (0.69)	0.96 (1.58)	1.22 (1.28)	1.06 (1.04)	1.13 (1.25)
Total	1.58 (1.60)	1.88 (1.96)	1.18 (1.55)	1.32 (1.28)	1.44 (1.28)	1.45 (1.56)
Middle adulthood events						
Early midlife	4.50_abcd_ (3.63)	2.17_a_ (2.29)	2.00_b_ (1.78)	1.81_c_ (1.57)	2.48_d_ (2.39)	2.36 (2.32)
Late midlife	1.67 (1.80)	3.15 (2.01)	2.35 (1.84)	2.22 (1.76)	2.61 (2.36)	2.52 (2.03)
Total	3.16 (3.18)	2.69 (2.18)	2.22 (1.80)	2.00 (1.65)	2.54 (2.37)	2.44 (2.18)
Neg LE totals						
Early midlife	7.60_abc_ (5.62)	5.65 (4.24)	3.85_a_ (2.58)	3.85_b_ (2.37)	5.02_c_ (3.32)	4.90 (3.57)
Late midlife	3.89 (2.67)	5.12 (3.27)	3.61 (2.39)	4.09 (2.31)	3.92 (2.52)	4.12 (2.65)
Total	5.84_a_ (4.75)	5.37_b_ (3.72)	3.71_ab_ (2.44)	3.96 (2.32)	4.53 (3.02)	4.51 (3.16)

Matching subscripts indicate significant post hoc results

*p < .05, **p < .01, ***p < .001

#### Life Event Valence

Five (resilient group) by two (midlife group) repeated measures MANOVA analyses were also conducted for the life event valence variables (positive, neutral, and negative). Due to the results of Mauchly’s sphericity test, Greenhouse-Geisser corrected statistics are provided (correction factor = .951). The within-subjects outcome for differently valenced life events was significant, *F* (2, 478) = 33.83, *p* ≤ .001, *η*^2^ = .124, due to the greater quantity of negative life events compared to positive and neutral. On average, participants reported 2.86 positive life events (*SD* = 2.46); 2.77 neutral life events (*SD* = 2.36) and 4.51 negative life events (*SD* = 3.16). The within-subjects interaction of life event valence categories and resilient group was also significant, *F* (8, 478) = 2.01, *p* = .047, primarily due to the increased reporting of negative life events by the low resilient group and by the religious group (*M* = 5.84, 5.37, *SD* = 4.75, 3.72, respectively), and the lower quantities for the medium and non-religious resilient groups (*M* = 3.71, 3.96, *SD* = 2.44, 2.32, respectively). This outcome is depicted in the last set of clustered bars in Fig. 2. None of the other within-subjects interactions were significant. The between subjects effect of midlife group was significant, *F* (1, 239) = 5.60, *p* = .019, *η*^2^ = .023, due to early midlife participants reporting more life events overall compared to late midlife participants (*M* = 10.66, 9.62; *SD* = 5.05, 4.69, respectively). The remaining effects and interactions were not significant.

#### Life Event Categories

Five (resilient group) by two (midlife group) analyses were also conducted for the life event category variables (death, relationship, work, finances, health, abuse, and trauma) individually due to large differences across categories. None of the main effects of resilience group were significant. The midlife group differences were observed for death, relationship, work, and finances, replicating the results presented in Table 6.

#### Summary

The analyses reported above suggest that resilient groups differ across variables measuring perceptions of future opportunities, recovery from hardship, overall life satisfaction, and persistence when faced with challenges. In addition, these results reveal that the *timing* of life events may not influence psychological resilience. However, it is noteworthy that moderate life event counts are associated with psychological benefits compared to low or high counts, replicating Seery et al. (2010).

### Predicting Life Satisfaction from Life Events and Psychological Resilience

Zero-order correlations between SWLS and the life event and resilience variables were generally significant (see column 1 in Table 10). The negative life event, resilience variables, educational degree, and age were entered in five steps: (1) life events; (2) MLS subscales; (3) other resilience measures; (4) education (years); (5) followed by age. It was hypothesized that each of these variable groups would contribute significantly to the SWL variance, with little if any unexplained variance remaining on the final step when age was entered. For the first step, the negative life event categories (death, relationship, work, finances, and health) accounted for 19.6% of the SWL variance, with significant coefficients for work and health (*B* = − .312, − .149, respectively). At the second step, the MLS subscales accounted for an additional 31.2% of the variance, for a total of 50.8%. Notably, the coefficients for growth, managing uncertainty, and humor were significant (*B* = .315, .233, .123, respectively). At this point, all but the coefficient for relationship hardships and SWL were significant. On the third step, FTP, BRS, and Grit were entered, adding a significant 2.0% variance, explaining 52.7% of the variance in life satisfaction. Only the BRS coefficient was significant (*B* = .148), and previously significant coefficients remained significant. The managing uncertainty coefficient dropped, suggesting collinearity. Education was entered on the fourth step, explaining a small but significant amount of variance: 2.0% with a significant *B* of .149, for a total of 54.8% variance explained. Entering age left the coefficients virtually unchanged. After this final step, variables predicting SWL included death, work, health, growth, managing uncertainty, FTP, BRS, and education. Among these, work negative life events and the growth MLS subscale yielded the greatest coefficients (*B* = .257, .255, respectively).

**Table 10:** Predictors of life satisfaction: resilience, life events, and age

	Zero-order correlation	1: Enter neg LE	2: Enter MLS vars	3: Enter res vars	4: Enter educ	5: Enter age
All Predictors		*B*	*B*	*B*	*B*	*B*
Death	.094	.095	.125**	.120**	.130**	.115*
Relationship	− .206***	− .054	− .039	− .039	− .021	− .017
Work	− .385***	− .312***	− .245***	− .241***	− .260***	− .257***
Finances	− .279***	− .110	− .132**	− .116*	− .105*	− .094
Health	− .169**	− .149**	− .144**	− .144**	− .133**	− .135**
Growth	.483***		.315***	.256***	.250***	.255***
Spirit	.115		− .030	− .031	− .028	− .035
Man unc	.507***		.233***	.127*	.108*	.107*
Humor	.386***		.123*	.102	.088	.093
Objectivity	.362***		.051	.008	− .009	− .015
FTP	.422***			.098	.100	.117*
BRS	.541***			.148*	.171*	.159*
Grit	.334***			.050	.034	.034
Educ	.204***				.149***	.149***
Age	.130*					.071
ΔR^2^		.196***	.312***	.019*	.020***	.004
*R*^2^		.196***	.508***	.527***	.548***	.552***
Without BRS						
Death		.095	.125**	.113**	.121**	.104*
Relationship		− .054	− .039	− .034	− .017	− .012
Work		− .312***	− .245***	− .257***	− .277***	− .273***
Finances		− .110	− .132**	− .121*	− .111*	− .098*
Health		− .149**	− .144**	− .142**	− .132**	− .134**
Growth			.315***	.275***	.272***	.276***
Spirit			− .030	− .039	− .038	− .045
Man unc			.233***	.174**	.164**	.158**
Humor			.123*	.117*	.107*	.110*
Objectivity			.051	.028	.032	.022
FTP				.117*	.121*	.139**
Grit				.071	.060	.058
Educ					.138**	.139***
Age						.083
ΔR^2^		.196***	.312***	.011*	.018**	.005
*R*^2^		.196***	.508***	.519***	.537***	.543***
Robust predictors						
Work		− .377***	− .313***	− .317***	− .330***	− .310***
Health		− .149**	− .138**	− .136**	− .120**	− .127**
Growth			.336***	.290***	.285***	.292***
Manag			.291***	.240***	.222***	.206***
FTP				.143**	.145**	.169**
Educ					.153***	.154***
Age						.128**
ΔR^2^		.171***	.283***	.014**	.023***	.015**
*R*^2^		.171***	.454***	.467***	.490***	.505***

*N* = 267. Standardized coefficients provided

*p < .05, **p < .01, ***p < .001

To examine multicollinearity issues, tolerance values (identifying the percentage of variance in each variable that is independent of the other predictors) were obtained. Most of the tolerance values were well above 50%, with the lowest values associated with BRS and managing uncertainty (.366; .451, respectively), suggesting that 36.1% of the variance in BRS and 45.1% of managing uncertainty are independent of other predictors. Overall, the results of the multicollinearity diagnostics suggest that all of these values are unproblematic (Cohen et al. 2003). However, because of the potential overlap between BRS and managing uncertainty, a second analysis without BRS is also provided in Table 10. This second analysis results in more stable results for MLS-managing uncertainty. Overall, it can be concluded that overall life satisfaction may be the result of experiencing particular life events (most robust here were work-related negative life events), as well as variables associated with resilience (particularly the sense of growth from past experiences), with little overlapping variance across these variables.

Table 10 also includes the regression analysis with the most robust predictors. For this analysis, work and health were entered first, then growth and managing uncertainty, then FTP, followed by education and age. All of these predictors were significant, explaining 50.5% of life satisfaction variance. Life satisfaction may be undermined in people who have experienced above average levels of negative work and/or health life events, but may increase when individuals feel that they have learned from their experiences or feel that they can manage some of life’s uncertain challenges. In addition, people who feel that they will have opportunities in the future have higher levels of life satisfaction.

### Comparing Predictors Across Early and Late Midlife Samples

Regression analysis was also used to examine the effects of negative life events, resilience factors, future time perspectives, education, and age on well-being for the two midlife groups separately, with results provided in Table 11. Overall, these variables predicted more of the variance in the late midlife group (59.9% of the variance, compared to 47.3%). The influence of work negative life events was impressive in the early midlife sample *(B* = .339) and smaller in the late midlife sample (*B* = − .281). Negative health life events had virtually no impact on early midlife participants, but had a significant impact for the older group (*B* = − .028; − .229, respectively). This result is probably the result of work-related challenges becoming less likely and less salient as middle-aged adults leave or prepare to leave the work force. Notably, MLS Growth scores had little impact on life satisfaction in the early midlife group (*B* = .122, not significant), but were related in the late midlife group (*B* = .478), suggesting that the sense of growth becomes more psychologically relevant as people approach and go through their 60s. FTP influenced life satisfaction for the early midlife group (*B* = .250), but not the late midlife group (*B* = .053), suggesting that concerns about future opportunities are more salient for early midlife adults. Educational degree attainment influenced life satisfaction in the late midlife group, but not in the younger group (*B* = .217; .143, respectively). It is possible that education attainment has a cumulative effect over time; or that this variable is a proxy for managing age-related challenges, thereby becoming more relevant as people go through their 60s. Age contributed a small amount of significant variance in the late midlife group, but not in the early midlife group, indicating that something associated with age had an influence, but primarily in the older group. These different configurations of predictors highlight developmental psychological changes affecting resilience and well-being that emerge as people go through early and late midlife.

**Table 11:** Robust predictors of life satisfaction in two midlife groups

	Zero-order correlation	1: Enter neg LE	2: Enter MLS scales	3: Enter FTP	4: Enter educ	5: Enter age
Early midlife, *N* = 134						
Work	− .442***	− .441***	− .328***	− .331***	− .331***	− .339***
Health	− .061	− .016	− .024	− .034	− .023	− .028
Growth	.406***		.164*	.121	.124	.122
Manag	.556***		.390***	.249**	.242**	.239**
FTP	.515***			.250**	.249**	.250**
Educ	.17				.083	.143
Age	− .035					.019
ΔR^2^		.196***	.234***	.034**	.009	.000
*R*^2^		.196***	.430***	.464***	.473***	.473***
Late midlife, *N* = 133						
Work	− .263**	− .264***	− .257***	− .258***	− .279***	− .281***
Health	− .280***	− .281***	− .250***	− .247***	− .227***	− .229***
Growth	.614***		.532***	.515***	.502***	.478***
Manag	.442***		.202**	.197**	.174**	.167**
FTP	.355***			.039	.040	.053
Educ	.275***				.182**	.217***
Age	.129					.122*
ΔR^2^		.148***	.406***	.001	.031**	.013*
*R*^2^		.148***	.554***	.555***	.586***	.599***

*p < .05, **p < .01, ***p < .001

### The Effects of Education on Negative Life Events and MLS-Adversity

**Fig. 3:**
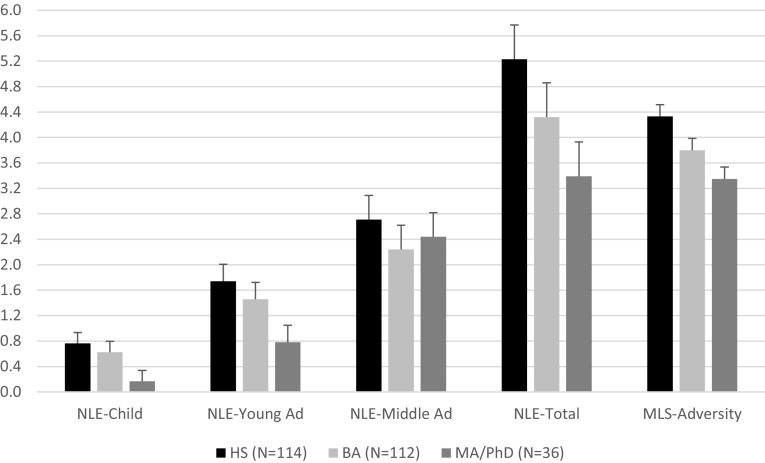
Negative life events by education groups. Note: *N* = 267. *NLE* negative life events. Error bars reflect standard error of the mean for each level of education

Three (Education: HS, BA, PhD) by three (NLE time frame: childhood, young adulthood and middle adulthood) repeated measures MANOVA analyses were conducted for negative life events totals, with education as a between subjects factor and NLE time frame as a within-subjects factor. There was, as expected, a within-subject effect of NLE time frame on NLE counts, with differences across event counts (e.g., more events in middle adulthood). The between subject main effect of education on NLE total counts was also significant, *F* (2, 264) = 5.55, *p* = .004, *η*^*2*^= .040. Post hocs revealed that high school degree participants significantly exceeded BA and MA/PhD participants, but the difference between BA and MA/PhD participants was not significant. The interaction of education and NLE time frame was not significant *F* (3.098, 401.2) = 1.36, n.s, suggesting that the effects of education on negative life events were similar across all three time frames. Descriptive and ANOVA statistics for the effects of educational degree on childhood, young adulthood, and middle adulthood negative life event counts are provided in Fig. 3. These results demonstrate that there were significant differences for childhood, young adulthood, and for total negative life events, as well as significant differences in total counts across educational degree attainment.

Education also had an impact on MLS adversity, *F* (2, 264) = 13.55, *p* ≤ .001, *η*^2^ = .093 (see Fig. 3). The effects of educational degree on MLS-adversity resembles the effect on negative life event counts, suggesting that these two variables, negative life event counts, and MLS adversity measure similar phenomenon.

Three (Education: HS, BA, PhD) by five (NLE category: death, relationships, work, finances, and health) repeated measures MANOVA analyses were conducted for negative life events totals, with education as a between subjects factor and NLE category as a within-subjects factor. There was, as expected, a within-subject effect of NLE category due to more counts associated with death. However, the between subject effect of education on NLE total was not significant, *F* (2, 259) = 2.76, *p* = .065. This pattern of almost significant was repeated for relationships, *F* (2, 264) = 2.90, *p* = .057, with significant post hoc differences for HS vs. MA/PhD; and for health: *F* (2, 259) = 2.54, *p* = .081 with significant post hoc differences for HS versus MA/PhD (HS > MA/PhD for both). Taken together, these education and negative life event results suggest that people with high school levels of education are more likely to report more negative life experiences overall, but that these experiences may occur at any age, or be of various types.

## Discussion

One of the goals of the current study was to develop and test a measure of resilience that included items about outcomes (e.g., growth, purpose) and processes (e.g., managing challenges, objectivity, and strategies). The Managing Life Survey (MLS) was administered in Study 1. Seven factors were identified: growth, managing uncertainty, spirituality, humor, emotional objectivity, humor, adversity, and changing philosophy. Support for this seven-factor solution was obtained in Study II (see Appendix). Taken together, these results provide support for using the MLS to assess aspects of resilience, such as growth or purpose, managing uncertainty, spiritual strategies, emotional objectivity, humor, and levels of adversity.

### Negative Life Events

A second goal pertained to exploring the valence and timing of life events more specifically than had been done previously, producing quantities reflecting negative life experiences during particular age ranges. Scores for negative life experiences were obtained using the modified Holmes and Rahe (1967) life event inventory. In this study, questions addressing valence and timing (e.g., childhood young adulthood) appeared when participants selected yes for negative events. Life events fell into seven categories: deaths, relationship, employment, finances, health issues, abuse, and trauma. The majority of the sample reported at least one negative death (74.5%) and at least one negative health event (54.7%).

Compared to late midlife participants, early midlife participants were more likely to report the following more often: divorces (46 vs. 26); loss of job (26 vs. 14); financial difficulties (66 vs. 37); and long-term unemployment (33 vs. 16). Even though relaxing attitudes toward divorce and economic declines may account for these, it is also possible that these are perceived as more negative because they are recent for this age group. Compared to early midlife, late midlife individuals reported more close family deaths (107 vs. 93), reflecting the greater likelihood of losing friends and relatives as people get older. They also reported more war trauma (12 vs. 0). Given the age range of the sample, this small group is most likely Vietnam veterans.

### Resilience Subgroups

Cluster and discriminant analysis was used to identify resilience subgroups, resulting in groups that were not only high and low, but that differed on reported endorsement of spiritual or religious strategies. For the five-cluster solution, eighty-four adults comprised the most resilient subgroup, generally scoring high on all of the MLS subscales. Just below the high resilient group was a group with similar MLS scores with the exception of low spirituality scores (*N* = 50). A third group had scores around the MLS subscale averages (*N* = 51). The remaining participants were below average, with one group below average on all MLS subscales except for MLS spirituality (*N* = 49). Scoring quite low across all subscales were nineteen individuals in the low resilient group. The adults in the low resilient group may have struggled substantially with the challenges they encountered. At the other end of the resiliency continuum were a large group of participants whose MLS subscale scores were all high, demonstrating that a sizable subset of individuals (*N* = 84) use all of these approaches (spiritual, humor, managing uncertainty) as well as endorsing items related to growth and purpose. Even though adults may vary in the approaches used to manage challenges, adults are usually able to manage and overcome challenges. The commonness of resilience in this midlife sample (*N* = 134 or 50.2% when high resilient and non-religious participants are combined) resembles the conclusion that resilience is typical in childhood and adolescence (Masten 2014).

It is noteworthy that MLS spirituality was the variable that differentiated in an unexpected manner. The high resilient group was likely to endorse spiritual strategies compared to the non-religious group (*M* = 5.26, 1.52, respectively). The religious group (*N* = 49) had the highest MLS subscale scores (*M* = 5.36), but below average scores on other MLS scales. Together, these results make it evident that many participants consider religious and/or spiritual strategies to be an important component of managing challenges; however, for a subset, spiritual strategies are not necessarily effective (Pargament 2011).

MLS Adversity scores declined significantly as resilience increased, but the lowest adversity levels were reported by the non-religious subgroup. Overall, individuals in the high and non-religious subgroups had higher levels of recovery (BRS); perseverance (Grit); future opportunities (FTP); and life satisfaction (SWL). These relationships provide convergent validity for the MLS as a measure of aspects of psychological resilience akin to growth, managing uncertainty, spirituality, and humor. In addition, the correlation of adversity and negative life events suggests that the MLS-adversity subscale may provide valid adversity scores, possibly precluding the need for a life event measure in future studies (*r* = .50 for MLS adversity and negative life event counts).

### Negative Life Events and Resilience

Negative life event counts were highest for the low resilient group (*N* = 19) and for the religious low-resilient group (*N* = 49), averaging 5.84 and 5.37 negative life events, respectively. The medium resilient group had the lowest negative life event counts, averaging 3.71 negative life events, but the non-religious average was close (3.96). The high resilient group averaged 4.53 negative life events. This trend is consistent with the idea that moderate levels of adversity may be associated with increases in overall psychological resilience, replicating Seery et al.’s (2010) results with different measures of life events and psychological well-being. It is clear that the high and the non-religious participants were not without hardship, but it is possible that they are managing adversity reasonably well. Some adversity and the successful management of that adversity may enhance skill and confidence in managing future adversity, but too much may overwhelm the development of resilience and well-being.

### Influence of Negative Life Events, Resilience, FTP, and Education on Well-Being

MLS growth, MLS managing uncertainty, and negative life events predicted 47.3% of the life satisfaction variance in the early midlife participants, and 59.9% of the life satisfaction variance in the late midlife participants. As might be expected, negative work experiences explained more of the variance in the early midlife group compared to late, and negative health experiences explained more variance in the late midlife group. Growth and purpose explained more life satisfaction variance in late midlife participants. Given that perceptions of growth and purpose may be based on evaluating psychological change after managing previous challenges, these evaluation processes may become gradually more salient as people go through midlife. In short, late midlife individuals may be more likely to consider how their unique experiences and the management of these have contributed to the person that they have become.

The effects of future time perspective were significant for early midlife participants, but not for late midlife (after controlling for negative life events, growth, and managing uncertainty). In early midlife, people who are aware of future opportunities may be more likely to view their lives as satisfying. However, as people get older and awareness of finitude increases, adults may transition to considering how their experiences have influenced them. Hence, as people transition into late midlife or early late life, growth from past experiences may have a larger influence on life satisfaction than previously, resulting in a shift from a future to a present orientation. In this manner, these data are consistent with perspectives on time orientation and its consequences as proposed by Socioemotional Selectivity theorists (Carstensen 2009).

Educational degree attainment was a more robust predictor in late midlife participants, explaining a small but significant amount of variance. Education may have a cumulative effect as people go through adulthood enhancing the management of challenges. It is possible that as challenges become more nuanced or complicated as people get older, with adults with lower levels of education find it difficult to manage these complex challenges (Gottfredson and Deary 2004). These results resemble findings that competence during one particular time frame in childhood or adolescence often leads to successful management of challenges at a later time (Masten 2014). It is possible that managing complex challenges in late midlife is influenced by competent management earlier in adulthood.

### Limitations

It is possible that computer users differ from non-users, potentially having more education and/or more income. Because the education levels were sufficiently variable in this sample, this may not have been the case. According to ongoing research, internet use has increased to 88% of the general population as of early 2017 (Pew Research Center 2017). In addition, AMT workers are usually trying to earn at least $10.00 an hour, so it seems unlikely that the sample overall disproportionally includes individuals from higher than average SES levels.

Even though Amazon Mechanical Turk (AMT) data have been found to resemble data obtained using other recruitment strategies, it is possible that there are differences, particularly given that middle-aged participants were recruited (Gosling et al. 2003; Paolacci and Chandler 2014). Overall, the results and psychometrics of the BRS, FTP, and life satisfaction scales resemble those obtained in previous studies suggesting that noise due to something amiss with this sample is possibly not a concern. At present, a non-AMT survey study with some of the same measures is underway: enabling a comparison in the future.

In addition, longitudinal approaches would improve the confidence that these results reflect developmental phenomenon. As such, this cross-sectional research is exploratory, setting the stage for longitudinal endeavors that have the potential to replicate the findings obtained herein.

### Contributions

Among the potential contributions of these two studies is the validation of the Managing Life Survey that addresses growth, managing uncertainty, spirituality, emotional objectivity, humor, and adversity in adulthood. The convergence with indicators of recovery, perseverance, adversity, and negative life event counts suggest that the MLS is a defensible assay of resilience. In addition, the use of MLS subscales to identify resilient subtypes suggests that resilience is not dichotomous (e.g., high, low), but that strategy preference also plays a key role. Two studies using MLS are underway: a study that includes adults in their 80s and 90s; and a study of LGBTQ adults.

The use and possible effectiveness of spiritual strategies varies across individuals. Two of the resilience groups identified in this study, the religious and the non-religious, could not be more different. The religious group had low scores on every measure of resilience, except spirituality, whereas the non-religious group had high scores on every measure, except spirituality. It is also noteworthy that these two groups, the religious and the non-religious, emerged across all cluster analyses, suggesting that this outcome is ecologically valid. In addition, the religious group had significantly higher scores on negative life event counts than the non-religious group. Given that the high resilient individuals had high scores on spirituality precludes concluding that religious or spiritual practices undermines resilience. Hence, it is not that religious or spiritual practices are necessarily ineffective. It is more likely that for some adults, this preference may reflect a tendency to use passive strategies. It is also possible that religious or spiritual approaches may be perceived as all that is available if challenges are considered insuperable.

This study replicates Seery et al.’s (2010) finding that moderate levels of adversity are more beneficial than the lowest and highest levels of adversity. The current study also suggests that some types of challenges may be more common or more salient at different ages. In early midlife, work-related challenges were negatively related to well-being, whereas health challenges negatively impacted well-being in late midlife. Even though at least one death was reported by over three quarters of the sample, death experiences did not impact well-being as much as work or health. Compared to other challenges, work and health challenges may be protracted, as well as intractable, increasing stressful levels of vigilance and frustration.

The current study also highlights a transition that may occur in midlife as awareness of getting older increases. Around these ages, work problems may begin to have less of an impact on well-being, whereas health problems have more. In addition, thoughts about future possibilities may become less salient; and perceptions of personal growth and purpose may become more salient. This result supports the hypothesis that midlife may be a pivotal time psychologically (Heckhausen 2001; Lachman et al. 2015; Ryff 1991). This pivot, more specifically, may pertain to a transition from “what am I going to do in the future” to “what have I done in the past.”

To some extent, the results of the current study resemble concepts related to wisdom, meaning making, and critical life events studied by developmental scientists interested in emerging adults (Webster 2013; Webster et al. 2017; Weststrate and Glück 2017). In that literature, critical life events may facilitate the development of wisdom in young adults whose self-reflective endeavors pertain to gaining substantial insight from these experiences (Webster 2003; Weststrate and Glück 2017). In the present study, participants with moderate levels of negative life events were more likely to have higher growth subscale scores with items resembling the meaning subscales in the emerging adulthood literature. In addition, participants with moderate levels of negative life events had higher managing uncertainty scores, which may correspond to self-reflective propensities. Given that the current study suggests that that growth and managing uncertainty processes vary across middle-aged adults in systematic ways, it may prove fruitful to examine the relationships among growth, managing uncertainty, wisdom, and self-reflection in a future study.

One fascinating aspect of adult development is that years of education acquired before the age of 25 has profound consequences several decades later. For example, education is correlated with both longevity and the avoidance of particular diseases (Gottfredson and Deary 2004). In this study, the most educated appear to have avoided above average levels of negative life events, and were more likely to find satisfaction in late midlife. University level education may prepare adults to keep learning in ways that benefits them psychologically and physically, such that the cumulative impact of education prepares people to manage the complexities and nuances of midlife and late adulthood.

## Conclusions

Overall this study demonstrates that psychological resilience is paramount in middle-aged adults, even though it manifests differently across subgroups. In addition, the adage that “What doesn’t kill us, makes us stronger” has some merit, in that adults may benefit from moderate levels of adversity (Seery et al. 2010). Managing life’s challenges consumes most adults as they go through adulthood, but at some point, reflection on growth and one’s purpose may become more salient as people approach late life, highlighting an important aspect of midlife psychology. Growth and purpose are critical areas of future research in midlife developmental psychology.

## Appendix: Managing Life Survey

Growth

1. Misfortunes present opportunities to become a different person than I was before. (7)
2. I tend to find personal growth in the hardships I have faced. (14)
3. When I face a difficult situation, I know I will learn something new. (5)
4. New experiences will foster continued personal development. (36)
5. In retrospect, most of the difficulties I have faced have NOT opened doors for me.* (1)
6. My ideas about my true purpose developed because of managing difficulties in the past. (21)
7. Usually I find that I change after dealing with new problems. (29)

Spirituality/religion as strategy

8. Praying or meditating is helpful when facing difficult situations. (24)
9. I do not rely on my spiritual or religious beliefs when experiencing hard times.* (16)
10. My spiritual or religious beliefs have helped me find my way through most difficult problems. (31)
11. I never ask for guidance by praying or meditating.* (6)

Adversity

12. I have faced a lot of hard times since I turned 18 years old. (35)
13. I faced a lot of hard times as a young adult. (4)
14. I have been bumped around by life. (32)
15. I faced a lot of hard times as a middle-aged adult (40–60). (23)

Managing uncertainty

16. I have a tendency to focus on too many aspects of a difficult situation.* (9)
17. I feel demoralized by my personal failures.* (28)
18. I have trouble accepting mistakes I have made when managing difficulties.* (22)
19. Some difficult situations are so complicated that I don’t know where to start.* (11)

Emotional objectivity

20. I can set aside my emotional reactions when solving life’s problems. (2)
21. I can set aside my emotional reactions when dealing with distressing situations. (30)

Changing philosophy

22. My ideas about what is important have stayed the same since I was about 20.* (10)
23. My ideas about what brings happiness in young adulthood have not changed.* (26)

Humor

24. Usually I cannot find anything humorous when dealing with misfortunes. * (18)
25. I often find humor in hardship or the way I react to hardship. (25)

Note: Numbers in parentheses indicate order on MLS as administered. Also, the adversity subscale could include an item appropriate for adults over 60: “I faced a lot of hard times as an older adult (60+).” *Reverse scored items.
